# Two Percentage of Ketoconazole Cream for the Treatment of Adult Female Acne: A Placebo-Controlled Trial

**DOI:** 10.7759/cureus.11581

**Published:** 2020-11-19

**Authors:** Ahsan Anwar, Syed Kamran Ul Hassan

**Affiliations:** 1 Dermatology, District Headquarters Teaching Hospital, Sahiwal, PAK

**Keywords:** adult female acne, ketoconazole, topical acne treatment.

## Abstract

Introduction

Our objective was to determine the efficacy of ketoconazole (KTZ) 2% cream for the treatment of mild adult female acne (AFA).

Materials and methods

This placebo-controlled trial was conducted in District Headquarters (DHQ) Teaching Hospital, Sahiwal, Pakistan. The study was completed in a period of January 2019-June 2020. A total of 60 females of age > 25 years having mild AFA were included. In Group I, the patients were advised to apply 2.0% KTZ cream covering the whole skin area twice daily for a period of eight weeks. In Group II (placebo group) patients, a topical cream containing propylene glycol was applied for similar period. After eight weeks, the cream was discontinued and participants were advised to use routine skincare products, and follow-up was done after two weeks. The main study outcome was reduction in the total count of acne lesions including both inflammatory and non-inflammatory lesions and overall success rate of treatment.

Results

Mean age was 36.2 ± 6.3 years in KTZ group versus 35.4 ± 6.5 years in control group. Mean duration of acne was 14.3 ± 7.3 years in KTZ group versus 15.1 ± 6.9 years in control group. Improvement in facial adult female acne scoring tool (AFAST) scale (AFAST-F) was observed in 13 (43.3%) patients in KTZ group and in only 4 (13.3%) patients in control group (p value = 0.009). Improvement in submandibular AFAST (AFAST-S) was observed in 12 (40.0%) patients in KTZ group and in eight (26.7%) patients in control group. The overall success rate of treatment was 14 (46.7%) in KTZ group versus 4 (13.3%) in control group (p value = 0.012).

Conclusions

In our study, we found significant improvement in reduction of acne lesions as well as complete recovery using 2.0% ketoconazole for the treatment of mild AFA. So KTZ can be used as a preferred treatment option for these patients.

## Introduction

Acne vulgaris is a very common skin disease particularly affecting the young females. It has significant psychological impact and impairs the daily living of affected ones [1]. It has been reported that more than twice of the women seek consultation from dermatologists in comparison to male patients, and more than one third of these visits are by women of age over 25 years [2,3]. Adult female acne (AFA) is defined as the onset of acne in females over the age of 25 years [4].

Hormonal, genetic, and environmental factors have been shown to be the main players in the pathogenesis of AFA. Among the environmental factors, stress, disturbed sleep, diet, excessive face washing, use of certain cosmetics, and exposure to ultraviolet radiation have been reported to be the triggering factors of AFA [5]. 

AFA can be in continuous or intermittent form and has reported to have high recurrence rates. The intermittent and recurrence nature of AFA has resulted in higher prevalence of antibiotic resistance. Therefore, long-term and continuous treatment is need is some cases that may continue even for years [6,7].

The commonly used treatment options for the management of AFA are benzoyl peroxide and adapalene [4]. These drugs are associated with skin irritation and are not effective against *Malassezia dysbiosis*. Ketoconazole (KTZ) cream has been shown to be effective for the treatment of AFA. KTZ is a broad spectrum azole antifungal, which was originally approved in 1981 for the treatment of systemic fungal infections. It has also been shown to have anti-inflammatory and anti-androgenic activity [8,9]. It has been shown to be effective against *Cutibacterium acnes*; it is also effective against resistant isolates [10].

Keeping in view the above-mentioned benefits of KTZ, the aim of the present study is to determine the efficacy of KTZ 2% cream for the treatment of mild AFA.

## Materials and methods

This placebo-controlled trial was conducted in District Headquarters (DHQ) Teaching Hospital, Sahiwal, Pakistan. The study was completed in a period of January 2019-June 2020. The ethical clearance approval for this study was taken from Ethical Review Committee (ERC) of hospital. The patients were informed about study protocol before taking informed consent. 

A total of 60 females of age > 25 years having mild AFA (AFA score 2, using the Global Acne Severity Scale) were included. Exclusion criteria were patients taking topic or systemic treatment for AFA for at least last two weeks, other facial skin lesions that can effect acne assessment, or pregnant females.

Patients were allocated to two equal groups using draw randomization. In Group I, the patients were advised to apply 2.0% KTZ cream covering the whole skin area twice daily for the period of eight weeks. In Group II (placebo group) patients, a topical cream containing propylene glycol was applied for similar period. After eight weeks, the cream was discontinued and participants were advised to use routine skincare products, and follow-up was done two weeks later. All the participants were advised to use skin-cleansing product based on nonmedical based ingredients such as sodium lauryl sulfate formulation before applying the cream. The follow-up was performed at two, four, six, and eight weeks; the final assessment was done at eighth week. A senior dermatologist who was unaware of the study protocol was asked to do final assessment of all patients.

The main study outcome was reduction in the total count of acne lesions including both inflammatory and non-inflammatory lesions and overall success rate of treatment. We used adult female acne scoring tool (AFAST) to evaluate face and submandibular areas separately. Acne improvement was defined as an improvement in acne severity score at least 1 point AFAST scale reduction from the baseline value, while the success rate was defined as achievement of 0 (clear) or 1 (almost clear) AFAST score at the end of follow-up.

Data analysis was done using SPSS v25 software (IBM Corp., New York). Acne improvement and success rate were compared using chi-square test between the KTZ and control group by taking p value ≤ 0.05 as a significant difference.

## Results

There was no significant difference regarding mean age, duration of acne, and family history between the group. Mean age was 36.2 ± 6.3 years in KTZ group versus 35.4 ± 6.5 years in control group. Mean duration of acne was 14.3 ± 7.3 years in KTZ group versus 15.1 ± 6.9 years in control group. There were 29 (96.6%) patients in KTZ group having persistent acne, while 27 (90.0%) patients in control group had persistent acne (p value = 0.3). The aggravating factors of acne were also similar between the groups (Table 1).

**Table 1:** Baseline characteristics. KTZ, Ketoconazole; AFA, adult female acne.

	KTZ Group (N = 30)	Placebo Group (N = 30)	p Values
Mean Age	36.2 ± 6.3	35.4 ± 6.5	0.63
Mean Duration	14.3 ± 7.3	15.1 ± 6.9	0.66
Family History of AFA	13 (43.3%)	12 (40.0%)	0.79
Type of AFA			
Persistent	29 (96.6%)	27 (90.0%)	0.3
Late-Onset	01 (3.33%)	03 (10.0%)	
Aggravating Factors			
Menstruation	23 (76.6%)	25 (83.3%)	0.51
Sunlight	09 (30.0%)	07 (23.3%)	0.55
Stress	03 (10.0%)	05 (16.6%)	0.45
Cosmetics	05 (16.6%)	03 (10.0%)	0.44
Disturbed Sleep	02 (6.67%)	01 (3.3%)	0.55

Improvement in facial AFAST scale (AFAST-F) was observed in 13 (43.3%) patients in KTZ group and in only four (13.3%) patients in control group (p value = 0.009). Improvement in submandibular AFAST (AFAST-S) was observed in 12 (40.0%) patients in KTZ group and in eight (26.7%) patients in control group. The overall success rate of treatment was 14 (46.7%) in KTZ group versus 4 (13.3%) in control group (p value = 0.012) (Table 2).

**Table 2:** Comparison of study outcomes between the groups. KTZ, Ketoconazole, AFAST-F, facial adult female acne scoring tool; AFAST-S, submandibular adult female acne scoring tool.

	KTZ Group (N = 30)	Control Group (N = 30)	p Value
Improvement in AFAST-F Scale			
Yes	13 (43.3%)	04 (13.3%)	0.009
No	17 (56.7%)	26 (86.7%)	
Improvement in AFAST-S Scale			
Yes	12 (40.0%)	08 (26.7%)	0.27
No	18 (60.0%)	22 (73.3%)	
Success Rate			
Yes	14 (46.7%)	05 (13.3%)	0.012
No	16 (53.3%)	25 (86.7%)	

## Discussion

In this placebo-controlled randomized trial we determined the efficacy of KTZ 2.0% cream for the treatment of mild AFA. We found significant improvement in the severity of AFA using KTZ, with overall success rate of 46.7% as compared to only 13.3% in control group.

Chottawornsak et al. conducted a similar study on the efficacy of KTZ for the treatment of AFA. The authors reported overall success rate of 45.0% in KTZ group and only 14.3% in control group. The study reported improvement in AFAST-F scale in 42.9% KTZ group patients and in 9.5% control group patients, and improvement in AFAST-S scale was reported in 35.0% KTZ group patients and in 33.3% control group patients [11].

In the present study, improvement in AFAST-F was observed in 43.3% patients in KTZ group and in 13.3% patients in control group, while improvement in AFAST-F scale was observed in 40.0% patients in KTZ group and in 26.7% patients in control group.

We chose the AFAST scale for evaluation of response rate of KTZ cream as this scale is more reliable for determining the improvements in acne severity [12,13]. Moreover, subjective evaluation of all patients by independent dermatologist (unaware of the study protocol) made the results more reliable.

Along with antifungal and anti-inflammatory properties, the other possible mechanisms of KTZ effectiveness may be due to its antilipase and anti-androgenic activity. Lipase is the most important enzyme for virulence activity of *C. acnes*; it produces inflammation and follicular hyperplasia in infected individuals [14]. Inhibition of lipase activity results in both decrease in free fatty-acid components in sebum and thus suppression of comedo formation. Moreover, systemic KTZ also inhibits androgen production by inhibiting the cytochrome P450-dependent enzymes in testis, adrenal glands, and ovaries [9]. So this can be hypothesized that the topical KTZ may possess similar effects by inhibiting steroidogenesis role of pilosebaceous unit [15].

KTZ also exhibits its antilipase activity against *Malassezia *spp., which also has lipase activity more than that of *C. acnes* [16]. Some researchers have reported that *Malassezia *spp. is responsible for recurrent acne and other skin inflammatory lesions such as seborrheic dermatitis [17]. As KTZ treatment is markedly effective in improving the acne lesions and is also effective against seborrheic dermatitis, the use of KTZ can help to treat both types of lesions that often present together.

The major limitation of present study is that we only included patients having mild degree of AFA; so the results of present study cannot be generalized to treat patients having moderate to severe acne. There is a need to conduct studies with large sample size including patients having moderate and severe AFA so that the efficacy of KTZ treatment can be determined in these patients as well.

## Conclusions

In our study, we found significant improvement in reduction of acne lesions as well as complete recovery using 2.0% ketoconazole for the treatment of mild AFA. So KTZ can be used as a preferred treatment option for these patients.
